# Identification of molecular subtypes based on chromatin regulator-related genes and experimental verification of the role of ASCL1 in conferring chemotherapy resistance to breast cancer

**DOI:** 10.3389/fimmu.2024.1390261

**Published:** 2024-04-25

**Authors:** Yilun Li, Xiaolu Yang, Cuizhi Geng, Yunjiang Liu, Tiantian Tang, Lina Zhang, Fei Liu, Meng Zhang, Jun Hao, Li Ma

**Affiliations:** ^1^ Department of Breast Disease Center, The Fourth Hospital of Hebei Medical University, Shijiazhuang, China; ^2^ Department of Pathology, Hebei Medical University, Shijiazhuang, China; ^3^ Research Center, The Fourth Hospital of Hebei Medical University, Shijiazhuang, China; ^4^ Department of Pathology, The Fourth Hospital of Hebei Medical University, Shijiazhuang, China

**Keywords:** breast cancer, chromatin regulators, *ASCL1*, molecular subtypes, chemotherapy resistance

## Abstract

**Objective:**

The aim of this study was to identify the molecular subtypes of breast cancer based on chromatin regulator-related genes.

**Methods:**

The RNA sequencing data of The Cancer Genome Atlas–Breast Cancer cohort were obtained from the official website, while the single-cell data were downloaded from the Gene Expression Omnibus database (GSE176078). Validation was performed using the Molecular Taxonomy of Breast Cancer International Consortium dataset. Furthermore, the immune characteristics, tumor stemness, heterogeneity, and clinical characteristics of these molecular subtypes were analyzed. The correlation between chromatin regulators and chemotherapy resistance was examined *in vitro* using the quantitative real-time polymerase chain reaction (qRT-PCR) and Cell Counting Kit-8 (CCK8) assays.

**Results:**

This study identified three stable molecular subtypes with different prognostic and pathological features. Gene Ontology, Kyoto Encyclopedia of Genes and Genomes, and protein–protein interaction analyses revealed that the differentially expressed genes were associated with disease processes, such as mitotic nuclear division, chromosome segregation, condensed chromosome, and specific chromosome region. The T stage and subtypes were correlated with the clinical features. Tumor heterogeneity (mutant-allele tumor heterogeneity, tumor mutational burden, purity, and homologous recombination deficiency) and tumor stemness (RNA expression-based stemness score, epigenetically regulated RNA expression-based stemness score, DNA methylation-based stemness score, and epigenetically regulated DNA methylation-based stemness score) significantly varied between the three subtypes. Furthermore, Western blotting, qRT-PCR, and CCK8 assays demonstrated that the expression of ASCL1 was positively correlated with chemotherapy resistance in breast cancer.

**Conclusion:**

This study identified the subtypes of breast cancer based on chromatin regulators and analyzed their clinical features, gene mutation status, immunophenotype, and drug sensitivity. The results of this study provide effective strategies for assessing clinical prognosis and developing personalized treatment strategies.

## Introduction

Recently, breast cancer (BC) has become a major health concern among women worldwide. According to the Global Burden of Disease report, BC accounts for 30% of all cancers in women (1). The cancer statistics published in the CA journal revealed that BC is the most prevalent cancer in women and that its incidence continues to increase (2). Molecular biology studies have demonstrated that BC can be divided into the following four subtypes based on the expression of specific molecular markers: luminal A, luminal B, human epidermal growth factor receptor 2 (HER2)-positive, and triple-negative BC subtypes (3). The prognosis of patients with BC has not improved although several novel tumor markers have been identified and various innovative treatment modalities have been developed. Previous studies have demonstrated that patients with metastatic BC are associated with poor prognosis with a median overall survival (OS) of approximately 10 months (4). Therefore, there is an urgent need to identify novel specific tumor biomarkers that can accurately predict the prognosis of BC.

Epigenetic alterations play a major role in the development and progression of cancer (5). Previous studies have suggested that epigenetic changes, including DNA methylation, histone modification, and chromatin remodeling, are potential targets for the precision treatment of BC (5–7). Furthermore, chromatin regulators (CRs) play an indispensable role in regulating epigenetic processes (8). CRs epigenetically modulate gene expression and chromatin structure in response to both endogenous and exogenous cues. Epigenetic studies have suggested that the dysregulation of CRs can reprogram the epigenetic map of chromatin, contributing to the occurrence of various diseases, including cancer. Based on their epigenetic roles, CRs can be primarily categorized into the following three groups: DNA methylation factors, histone modifiers, and chromatin remodeling factors (9). These three groups of CRs interact with each other and are correlated with biological processes. The dysregulation of CRs is reported to be associated with diverse biological processes, such as inflammation, apoptosis, autophagy, and cell proliferation (10). In BC, CRs induce post-translational modification to activate estrogen receptor (ER), modulating the development and progression of ER-positive BC (11).

CR-related genes are closely associated with drug sensitivity. The CR-mediated alterations in the plasticity of chromatin promote tumor heterogeneity, resulting in the development of drug resistance in tumors (12). The effects of CRs on drug sensitivity in BC and the underlying mechanisms are unclear and must be elucidated.

In this study, the CRs associated with the prognosis of BC were identified using differential analysis and Cox analysis. Based on consistent clustering results, stable molecular subtypes with diverse prognostic and pathological features were identified. Furthermore, the immune characteristics, tumor stemness and heterogeneity, clinical characteristics, and functional pathways of different molecular subtypes were analyzed. Differential analysis was performed to identify the most significant differentially expressed genes (DEGs) in different molecular subtypes and explore their relationship with drug sensitivity. Finally, the core gene *ASCL1* was selected for *in vitro* validation.

## Materials and methods

### Data collection and identification of CR-related genes

The RNA sequencing data of The Cancer Genome Atlas–Breast Cancer (TCGA-BRCA) cohort were obtained from the official website (https://portal.gdc.cancer.gov/) and converted into transcripts per million formats. Additionally, the clinical follow-up, survival, and staging data were downloaded. To reduce bias in the statistical analysis, the data of patients with BC whose OS data were missing or had a follow-up time of <30 days and male patients with BC were excluded. Tumor mutational burden (TMB) and mutant-allele tumor heterogeneity (MATH) scores were calculated. CR-related genes were retrieved from the literature (10). Differentially expressed CRs were identified using the limma package in R software based on the following criteria: |log fold change (logFC)| >1 and false discovery rate (FDR) < 0.05 (13).

### Prognostic and enrichment analysis

The enrichment of differentially expressed CRs in the following Gene Ontology (GO) terms was determined: molecular functions, biological processes, and cellular components. Additionally, the enrichment of differentially expressed CRs in molecular pathways was determined using Kyoto Encyclopedia of Genes and Genomes (KEGG) analysis. The “clusterProfiler” package was used to perform GO and KEGG analyses (14). The GeneMANIA website (http://genemania.org/) was used to construct the protein–protein interaction (PPI) network. The prognostic values of DEGs were determined using univariate Cox regression analysis. The “Survminer” and “Survival” packages were used to perform survival analysis.

### Genotyping analysis and characterization of CR-related genes

Based on the expression of CR-related prognostic mRNA, a dimensional reduction of mRNA genotypes was performed using K-means clustering analysis with the “ConsensusClusterPlus” R package (15). Samples were divided into k clusters in which each sample belonged to the cluster with the most similar mean. To determine the construct validity of the genotyping clusters, the Kaplan–Meier log-rank test was used to compare survival between the clusters. Gene expression in different clusters was presented as a heatmap.

### Analysis of the correlation between immune characteristics among different clusters

Lists of genes encoding immune checkpoints and immunomodulators were downloaded from an integrated repository portal for tumor-immune system interactions (TISIDB, http://cis.hku.hk/TISIDB/download.php). Tracking Tumor Immuno-phenotype (http://biocc.hrbmu.edu.cn/TIP/) was used to calculate the immunoactivity score in different clusters. The pathway activity score was downloaded from the Gene Set Cancer Analysis website (GSCA, http://bioinfo.life.hust.edu.cn/GSCA/#/). The “CIBERSORT” package was used to evaluate the degree of tumor immune cell infiltration in different clusters. Gene expression, immune checkpoints, immunomodulators, and pathway activity scores in different subtypes were compared using the Kruskal–Wallis test. All results were visualized using the “ggplot2” and “ggpubr” packages.

### Analysis of gene mutation, tumor stemness, tumor heterogeneity, and clinical features of different clusters

TMB and MATH were used to predict the efficacy of tumor immunotherapy and tumor heterogeneity, respectively. The “maftools” package was used for calculating TMB and MATH scores and visualizing gene mutation in BC (16). The microsatellite instability (MSI), Neo, and purity scores were retrieved from the literature (17, 18). Six tumor stemness scores [RNA expression-based stemness score (RNAss), epigenetically regulated RNA expression-based stemness score (EREG-EXPss), DNA methylation-based stemness score (DNAss), epigenetically regulated DNA methylation-based stemness score (EREG-METHss), differentially methylated probe-based stemness score (DMPss), and enhancer element stemness score (ENHss)] were retrieved from the literature (19). Tumor stemness scores in different clusters were compared using the Kruskal–Wallis test. The chi-square method was used to analyze the clinicopathological characteristics of different clusters All results were visualized using the “ggplot2,” “ggpubr,” and “ggalluvial” packages.

### Identification of DEGs and the related pathways between three clusters

The following three pairs of clusters were analyzed to identify the DEGs: C3 and non-C3, C2 and non-C2, and C1 and non-C1. The R software gene set variance analysis package was used to examine the pathway score with the parameter set as method = “ssgsea” (20). “Estimate” and “ssgsea” methods were used to perform immune-related analysis. Correlation analysis was used to analyze the relationship between DEGs and pathway scores. The most significant DEGs were selected for the subsequent experiment. The correlation between immune cell infiltration and clinical characteristics was performed to explore the relationship between gene expression, immune cell infiltration, and clinical characteristics.

### Analysis of DEGs among three clusters using single-cell data

The single-cell data used in this study were exclusively obtained from tumor tissues. Furthermore, the Seurat R toolkit (v4.0.6) was used for processing single-cell data for dimensionality reduction, clustering of cell subspecies, and visualization (21). The data of cells were excluded based on the following criteria: (I) cells with >10% of mitochondrial genes; (II) cells with <500 transcripts per cell. The data were log-transformed, and the top 2,000 variable genes were selected as the input features of the principal component analysis (PCA). The dimensionality reduction was performed using a PCA matrix with 50 components using the RunPCA function of Seurat. The Uniform Manifold Approximation and Projection (UMAP) algorithm was used to further perform dimensionality reduction of the data, while the RunUMAP function was used to visualize the cluster. The function FindAllMarkers was used to identify unique marker genes for different cell types and label different cell types using classical cell markers. The dotplot displayed the section dedicated to the marker gene section. The expression of DEGs between three clusters was examined using the Featureplot function.

### Experimental materials

The Cell Counting Kit-8 (CCK8) detection kit was purchased from MedChemExpress, USA. The CellTiter 96® Aqueous One Solution Reagent Kit for cell viability detection was obtained from Promega Co. (Madison, WI, USA). Lipofectamine 3000 was purchased from Invitrogen Co. (Carlsbad, CA, USA). The ASCL1 expression plasmid with the green fluorescent protein tag (pGensil-1-ASCL1) was designed by Sangon Biotech Co. Anti-ASCL1, anti-β-actin, anti-ABCC1, and anti-ABCG2 antibodies were purchased from Hua’an Biologic Co.

### Cell culture

MCF7 cells were purchased from the Cell Resource Center, Institute of Basic Medicine, Chinese Academy of Medical Sciences and Peking Union Medical College and cultured in Dulbecco’s modified Eagle’s medium (DMEM) (4.5 g glucose, GIBCO) supplemented with 10% fetal bovine serum (GIBCO) and 1% penicillin/streptomycin (GIBCO). MCF7-ADR and MCF7-Taxol cells were purchased from Bena Culture Collection Co. and Ya’an Biology Co. (Guangzhou, China), respectively, and cultured in Roswell Park Memorial Institute-1640 (RPMI-1640) medium (GIBCO) supplemented with 10% fetal bovine serum (GIBCO) and 1% penicillin/streptomycin (GIBCO). All cells were cultured at 37 °C in a 5% CO_2_ humidified incubator.

### Cell transfection

MCF7, MCF7-ADR, and MCF7-Taxol cells were seeded in six-well plates and cultured to achieve 90% confluency. The cells were transfected with the indicated plasmid using Lipofectamine 3000, following the manufacturer’s instructions. Briefly, 2.5 μg of plasmid was mixed with 5 μL of P3000 in 125 μL of serum-free DMEM or RPMI-1640 medium. Meanwhile, 5 μL of Lipofectamine 3000 was mixed in 125 μL of serum-free DMEM or RPMI-1640 medium. These two mixtures were combined and incubated for 15 min at room temperature before addition to cells. The transfection efficiency was evaluated at 48 h post-transfection under an inverted fluorescence microscope.

### Quantitative real-time polymerase chain reaction analysis

Total RNA was extracted from MCF7, MCF7-ADR, and MCF7-Taxol cells using TRIzol reagent (Takara, Dalian, China). The isolated RNA was reverse-transcribed into complementary DNA (cDNA) using the reverse transcription kit (Takara), following the manufacturer’s instructions. Specific primers were used for amplification with cDNA as a template. The specific primers were as follows: Forward: CAAGCAAGTC AAGCGACAGC; Reverse: TTGACCAACTTGACGCGGTT. Based on the Ct value, the expression of the target gene was calculated using the 2^−ΔΔCT^ method.

### Western blotting analysis

Cells were lysed in radioimmunoprecipitation assay lysis buffer on ice. Equal amounts (20 μg) of proteins were subjected to sodium dodecyl sulfate-polyacrylamide gel electrophoresis using a 10% gel. The resolved proteins were transferred to 0.2-μm and 0.45-μm polyvinylidene difluoride membranes. The membranes were blocked with 5% skim milk powder for 1 h and incubated with primary antibodies (diluted 1:1,000) overnight at 4°C. The membranes were washed thrice with Tris-buffered saline containing 0.05% Tween-20 (TBST) (10 min/step). Immunoreactive signals were developed using a chemiluminescence solution. The protein gray values were analyzed using ImageJ software. The expression of the target protein was calculated using ACTB as the reference protein.

### CCK8 assay

Cells in the logarithmic phase were selected to prepare cell suspension. MCF7-ADR (5×10^3^ cells/100 µL) and MCF7-Taxol cells (5×10^3^ cells/100 µL) were seeded in 96-well cell culture plates and cultured for 24 h at 37°C. Next, the MCF7-ADR cells were incubated with drugs at concentrations of 0, 5, 30, 50, 80, and 100 mg/L, while the MCF7-Taxol cells were incubated with drugs at concentrations of 0, 5, 10, 20, 25, and 50 mg/L. At 48 h post-treatment, cells were incubated with 10 µL of CCK8 reagent for 2 h. The absorbance of the samples at 450 nm was measured. The inhibition rate was calculated as follows: inhibition rate = (1 − experimental group absorbance value/control group absorbance value). The half-maximal inhibitory concentration (IC_50_) value was calculated.

### Statistical analysis

Means between two groups were compared using the *t*-test, while those between more than two groups were compared using the Wilcoxon rank-sum and Kruskal–Wallis tests. Univariate Cox regression and chi-squared tests were used to assess the differences between count data. The log-rank test was used to evaluate the prognosis of cancer. Differences were considered significant at *p* < 0.05.

## Results

### DEGs and potential function of CRs in BC

**Figure 1:**
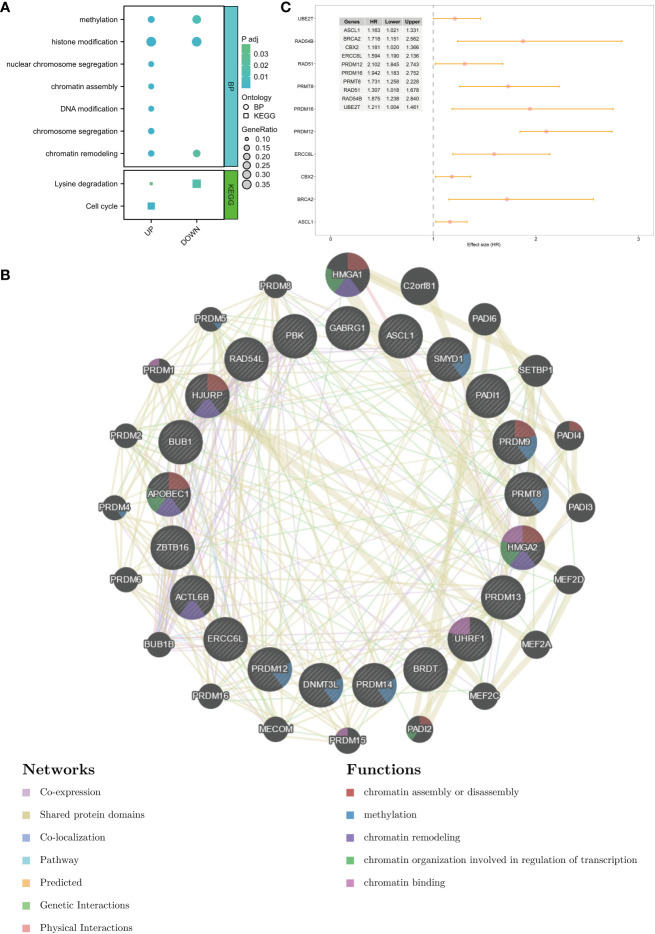
Identification of DEGs and potential function of chromatin regulators in breast cancer. **(A)** GO terms and KEGG pathways in which the DEGs are enriched. **(B)** PPI network of DEGs. **(C)** Prognosis-related genes were screened using univariate Cox analysis. DEGs, differentially expressed genes; GO, Gene Ontology; KEGG, Kyoto Encyclopedia of Genes and Genomes; PPI, protein–protein interaction.

In total, 65 differentially expressed CRs (14 downregulated CRs and 51 upregulated CRs) were identified between healthy breast tissues and BC tissues in the TCGA-BRCA cohort (**Supplementary Figure S1**). GO and KEGG enrichment analyses revealed that most upregulated CRs were closely related to methylation, histone modification, nuclear chromosome segregation, chromatin assembly, DNA modification chromosome segregation, chromatin remodeling, lysine degradation, and cell cycle (**Figure 1A**). Meanwhile, the downregulated CRs were associated with methylation, histone modification, chromatin remodeling, lysine degradation, and cell cycle (**Figure 1A**). PPI network analysis demonstrated that DEGs were associated with chromatin assembly or disassembly, methylation, chromatin remodeling, and chromatin organization involved in the regulation of transcription (**Figure 1B**). Univariate Cox analysis was performed to identify DEGs related to the prognosis of BC. The following 10 genes exhibited prognostic significance in patients with BC: *ASCL1*, *BRCA2*, *CBX2*, *ERCC6L*, *PRDM12*, *PRDM16*, *PRMT8*, *RAD51*, *RAD54B*, and *UBE2T* (**Figure 1C**). These genes were selected for subsequent analyses.

### Identification and validation of different molecular subtypes

**Figure 2:**
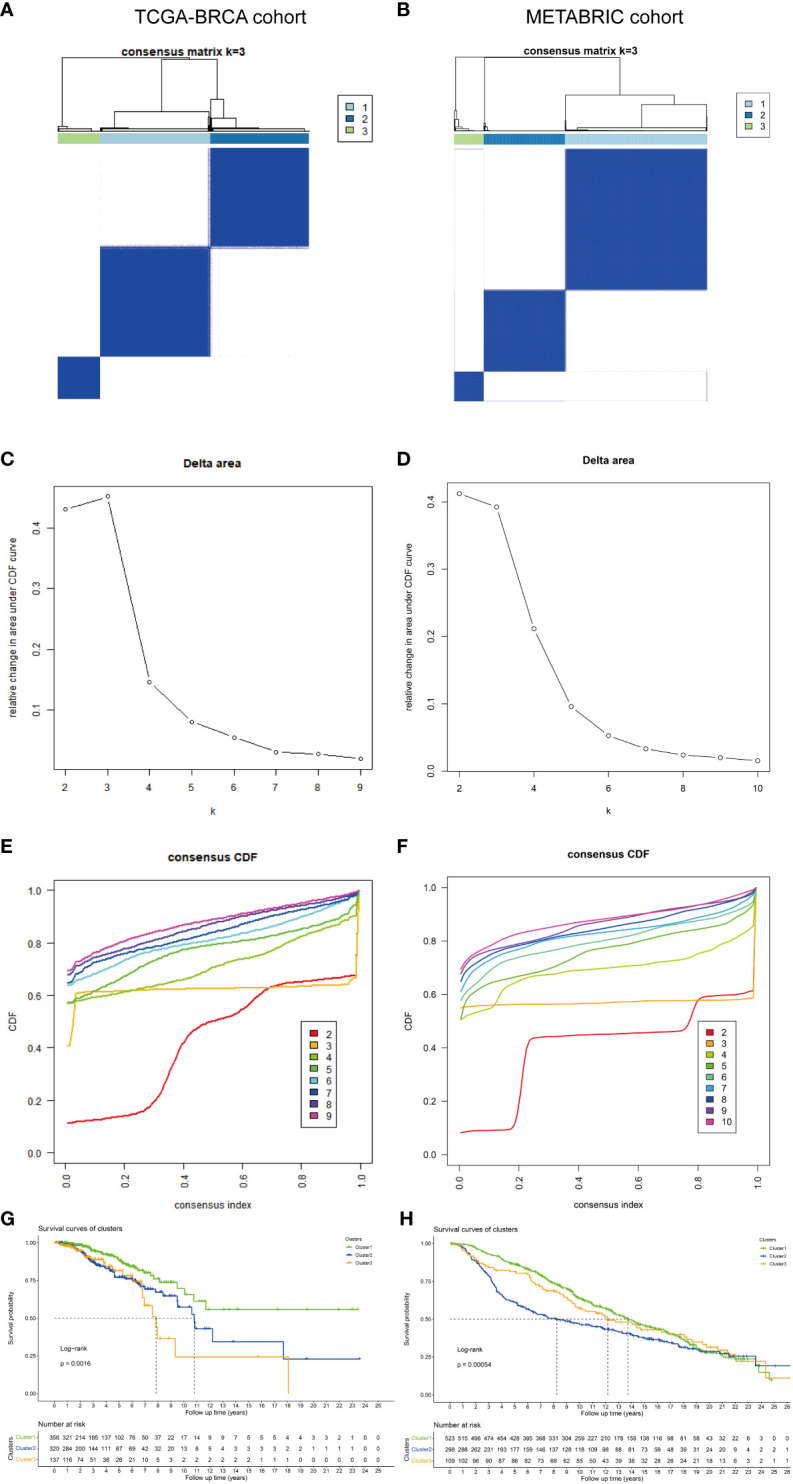
Identification and validation of different molecular subtypes. The consensus matrix revealed patients with three distinct CRG-related subtypes in **(A)** TCGA and **(B)** METABRIC datasets. Relative change in area under the CDF curve in **(C)** TCGA and **(D)** METABRIC datasets. Area under the CDF curve of a consensus matrix in **(E)** TCGA and **(F)** METABRIC datasets. Kaplan–Meier overall survival curves of patients with different subtypes determined based on CRGs (log-rank test) in **(G)** TCGA and **(H)** METABRIC datasets. DEGs, differentially expressed genes; CRGs, chromatin regulator-related genes; TCGA, The Cancer Genome Atlas; METABRIC, Molecular Taxonomy of Breast Cancer International Consortium; CDF, cumulative distribution function.

To identify the subtypes, a consensus clustering analysis was performed to categorize TCGA-BRCA samples based on the expression profiles of 10 CR-related genes. The METABRIC dataset was used for validation. The cumulative distributive function (CDF) delta area analysis indicated that a relatively stable clustering effect was achieved when *k* was equal to 3 in TCGA-BRCA (**Figures 2A, C**) and METABRIC cohorts (**Figures 2B, D**). Thus, three subtypes were identified in TCGA-BRCA (**Figure 2E**) and METABRIC cohorts (**Figure 2F**). The Kaplan–Meier curves demonstrated that the survival outcomes significantly varied between the three subtypes with cluster 1 exhibiting the most favorable prognosis in TCGA (*p* = 0.0016, **Figure 2G**) and METABRIC cohorts (*p* = 0.00054, **Figure 2H**). The DEGs between the three subtypes in two cohorts were represented using heatmaps (**Supplementary Figure 2**).

### Immune characteristics and potential functions of different subtypes

**Figure 3:**
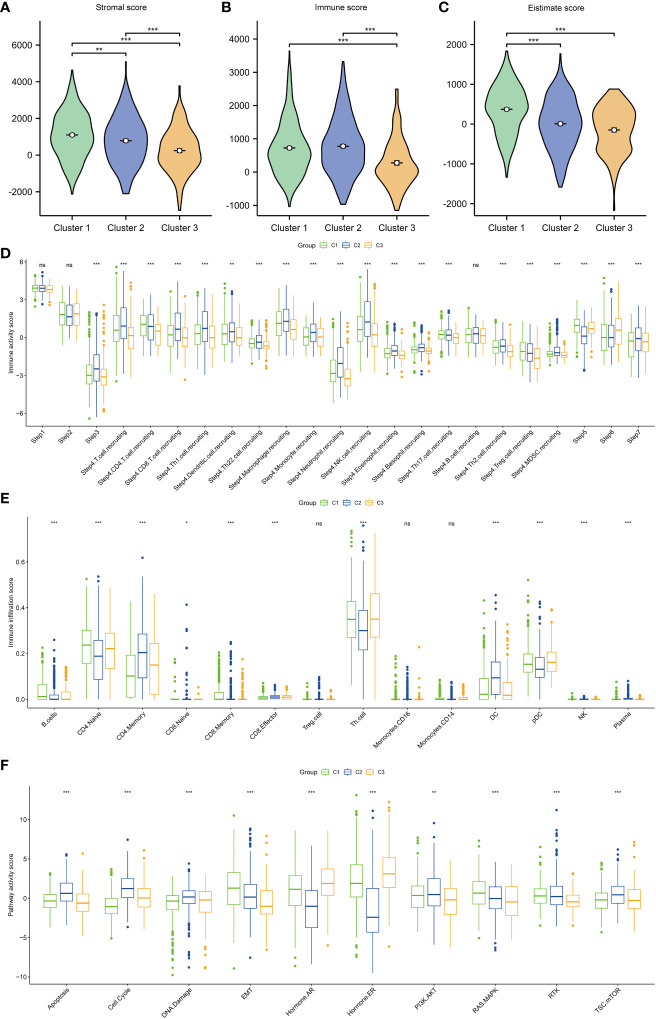
Immune scores and pathway activities of the three subtypes. **(A)** Stromal score in the three subtypes. **(B)** Immune score in the three subtypes. **(C)** Estimate score in the three subtypes. **(D)** Immune activity score in the three subtypes. **(E)** Immune cell infiltration score in the three subtypes. **(F)** Pathway score in the three subtypes. ^***^ *p* < 0.001; ^**^ *p* < 0.01; ^*^ *p* < 0.05.

Next, the immune characteristics and potential functions of different subtypes were examined. The stromal, immune, and estimate scores varied between the subtypes. Compared with those in cluster 1, the stromal, immune, and estimate scores were significantly lower in cluster 3 (**Figures 3A–C**). Analysis of the correlation between immune activity and different subtypes demonstrated that the immune activity scores in cluster 3 were lower than those in clusters 1 and 2, especially in terms of immune cell recruitment activities (**Figure 3D**). Additionally, the proportions of B cells, CD4 cells, CD8 cells, T helper (Th) cells, dendritic cells (DCs), natural killer (NK) cells, and plasma were varied between the subtypes (**Figure 3E**). Analysis of the activity of pathways in different subtypes revealed that apoptosis, cell cycle, DNA damage, epithelial-to-mesenchymal transition (EMT), ER and AR, PI3K-AKT, RAS-MAPK, RTK, and TSC-mTOR varied between the subtypes (**Figure 3F**).

The expression of immune checkpoint and immunomodulators in different subtypes was analyzed (**Supplementary Figure S3A**). Immunomodulator inhibitor-related (**Supplementary Figure S3B**), major histocompatibility complex (MHC)-related (**Supplementary Figure S3C**), receptor-related (**Supplementary Figure S3D**), immunomodulator stimulator-related (**Supplementary Figure S3E**), and chemokine-related genes (**Supplementary Figure S3F**) were differentially expressed in the three clusters. Furthermore, immune checkpoint pathway-related genes exhibited differential expression in the three clusters (**Supplementary Figures S3G, H**).

### Tumor heterogeneity, stemness, and mutation in different subtypes

**Figure 4:**
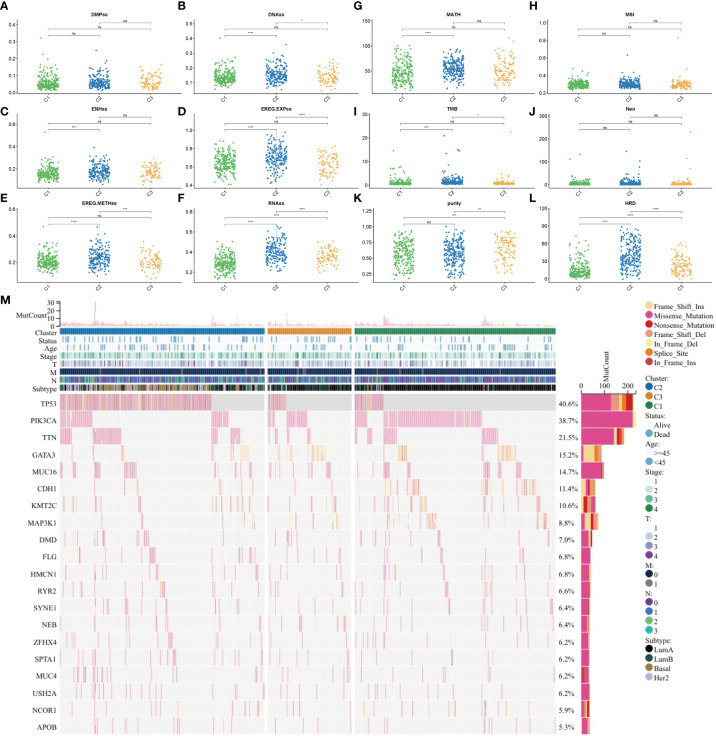
Tumor heterogeneity, stemness, and mutation in different subtypes. **(A)** DMPss, **(B)** DNAss, **(C)** ENHss, **(D)** EREG-EXPss, **(E)** EREG-METHss, and **(F)** RNAss in different subtypes. **(G)** MATH, **(H)** MSI, **(I)** TMB, **(J)** Neo, **(K)** purity, and **(L)** HRD scores in different subtypes. **(M)** Gene mutation in different subtypes. ****p<0.0001; ***p < 0.001; **p < 0.01; *p < 0.05; ns: no significance. DMPss: differentially methylated probe-based stemness score; DNAss, DNA methylation-based stemness score; ENHss, enhancer element stemness score; EREG-EXPss, epigenetically regulated RNA expression-based stemness score; EREG-METHss, epigenetically regulated DNA methylation-based stemness score; RNAss, RNA expression-based stemness score. MATH, mutant-allele tumor heterogeneity; TMB, tumor mutational burden; MSI, microsatellite instability; HRD, homologous recombination deficiency.

To examine the tumor heterogeneity and mutation profiles in different subtypes, the indicators DMPss, DNAss, ENHss, EREG-EXPss, EREG-METHss, and RNAss (**Figures 4A–F**), as well as MATH, MSI, TMB, Neo, purity, and homologous recombination deficiency (HRD) scores (**Figures 4G–L**), were analyzed. The RNAss, EREG-EXPss, DNAss, and EREG-METHss were significantly different between the three subtypes. Additionally, the MATH, TMB, purity, and HRD scores varied between the three subtypes. *TP53*, *PI3KCA*, *TTN*, *GATA3*, and *MUC16* were significantly mutated in the three subtypes (**Figure 4M**).

### Clinical characteristics of the three subtypes

Next, the clinical characteristics of the three different subtypes were examined. A Sankey diagram was used to visualize the total characteristics of the three clusters (**Supplementary Figure S4A**). T and tumor stages were closely related to the three subtypes. T1, stage 1, and luminal A subtypes were enriched in cluster 1 (**Supplementary Figures S4C, F, G**). However, other clinical characteristics (N, M, and age) were not significantly different between the three clusters (**Supplementary Figures S4B, D, E**).

### Enrichment of DEGs between the three clusters in pathways

**Figure 5:**
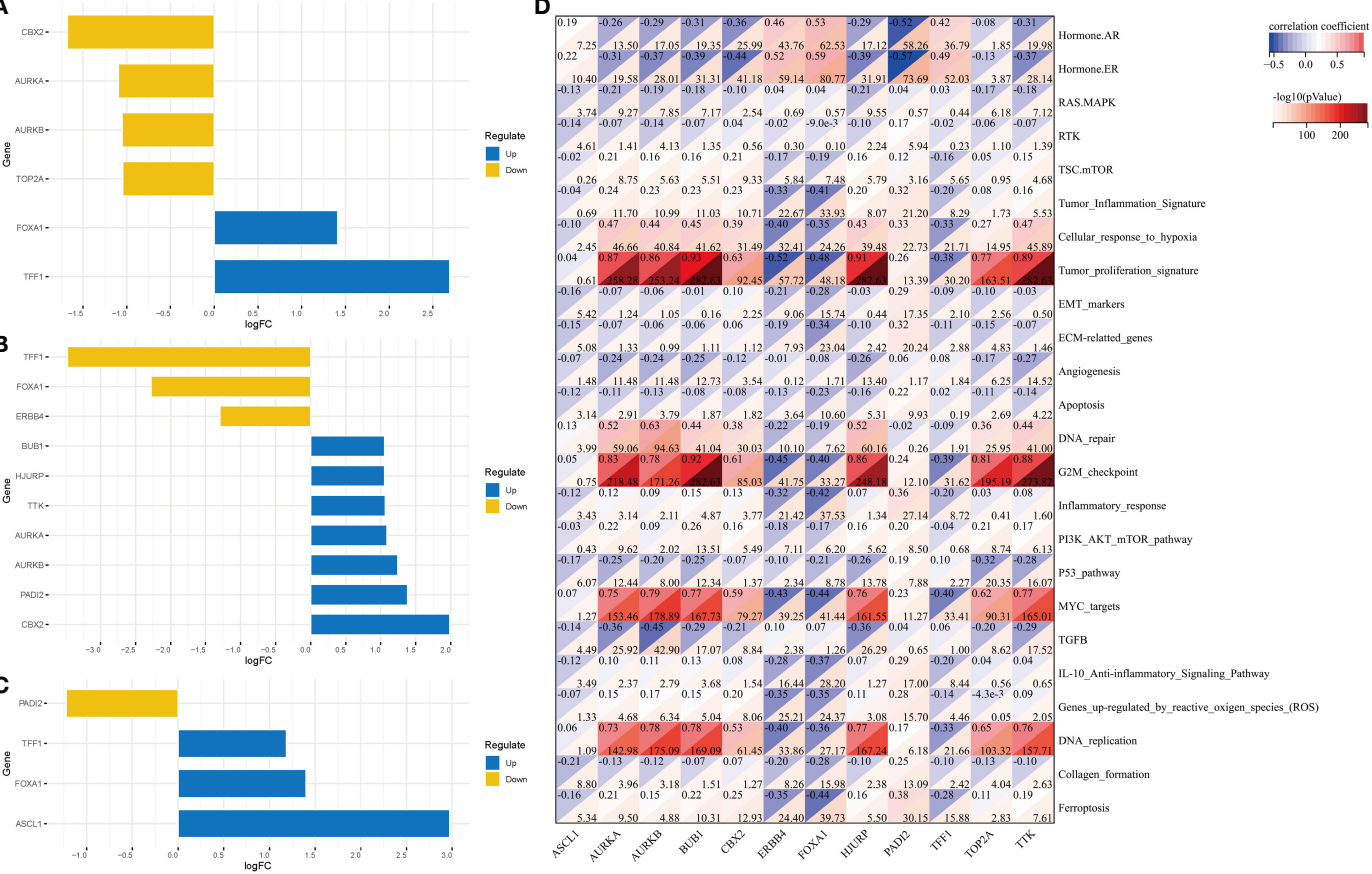
DEGs in the three clusters and their related pathways. DEGs between the following pairs: **(A)** C1 and non-C1 groups; **(B)** C2 and non-C2 groups; **(C)** C3 and non-C3 groups. **(D)** Correlation analysis revealed the pathways in which DEGs are enriched. DEGs, differentially expressed genes.

The following three subtype pairs were compared to identify DEGs: C1 and non-C1 groups, C2 and non-C2 groups, and C3 and non-C3 groups. Compared with those in the non-C1 group, the *CBX2* expression levels were significantly downregulated and the *TFF1* expression levels were upregulated in the C1 group (**Figure 5A**). Meanwhile, compared with those in the non-C2 group, the *TFF1* expression levels were significantly downregulated and the *CBX2* expression levels were significantly upregulated in the C2 group (**Figure 5B**). Additionally, compared with those in the non-C3 group, the *PADI2* expression levels were significantly downregulated and the *ASCL1* expression levels were significantly upregulated in the C3 group (**Figure 5C**). The most significant gene (*ASCL1*) was selected for subsequent analyses. Correlation analysis revealed a significant correlation of *AURKA*, *AURKB*, *BUB1*, *HJURP*, *TOP2A*, and *TTK* with tumor proliferation, G2M checkpoint, MYC targets, and DNA replication (**Figure 5D**).

### Single-cell data analysis of the distribution and expression of DEGs in various clusters

**Figure 6:**
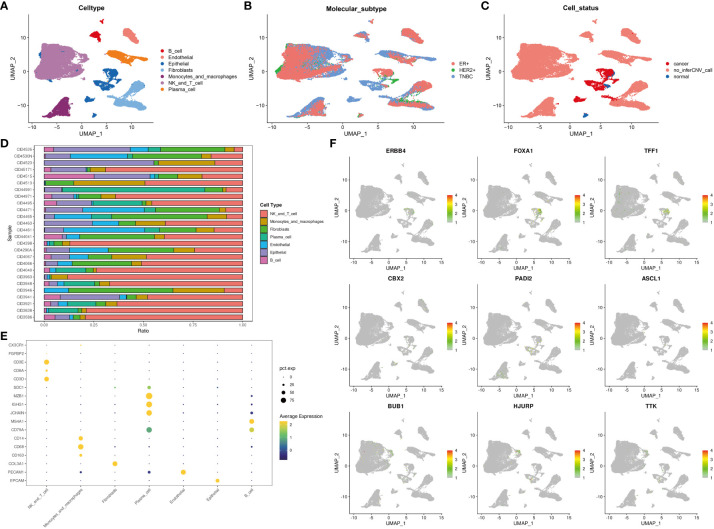
The expression and distribution of DEGs among different clusters based on single-cell data analysis. The distribution of different cell types **(A)**, different molecular subtypes **(B)**, and different cell statuses **(C)**. **(D)** The proportion of different cell types in samples. **(E)** Cell markers were displayed using “dotplot.” **(F)** The expression and distribution of DEGs among different clusters were analyzed using the “Featureplot” package. DEGs, differentially expressed genes.

Single-cell data were used to show the distribution of different cell types (**Figure 6A**), molecular subtypes (**Figure 6B**), and cell statuses (**Figure 6C**). The distribution of various cell types in different samples was also examined (**Figure 6D**). Additionally, the expression of various cell markers was analyzed (**Figure 6E**).

Analysis of gene expression and distribution at the single-cell level (**Figure 6F**) revealed that *ERBB4*, *FOXA1*, *TFF1*, *CBX2*, *PADI2*, *ASCL1*, *BUB1*, *HJURP*, and *TTK* were expressed in tumor cells. Meanwhile, *BUB1*, *HJURP*, and *TTK* were expressed in T cells or NK cells. These DEGs can potentially contribute to the functions of tumor cells, T cells, or NK cells.

### ASCL1 is significantly correlated with the clinical characteristics and immune cell infiltration

**Figure 7:**
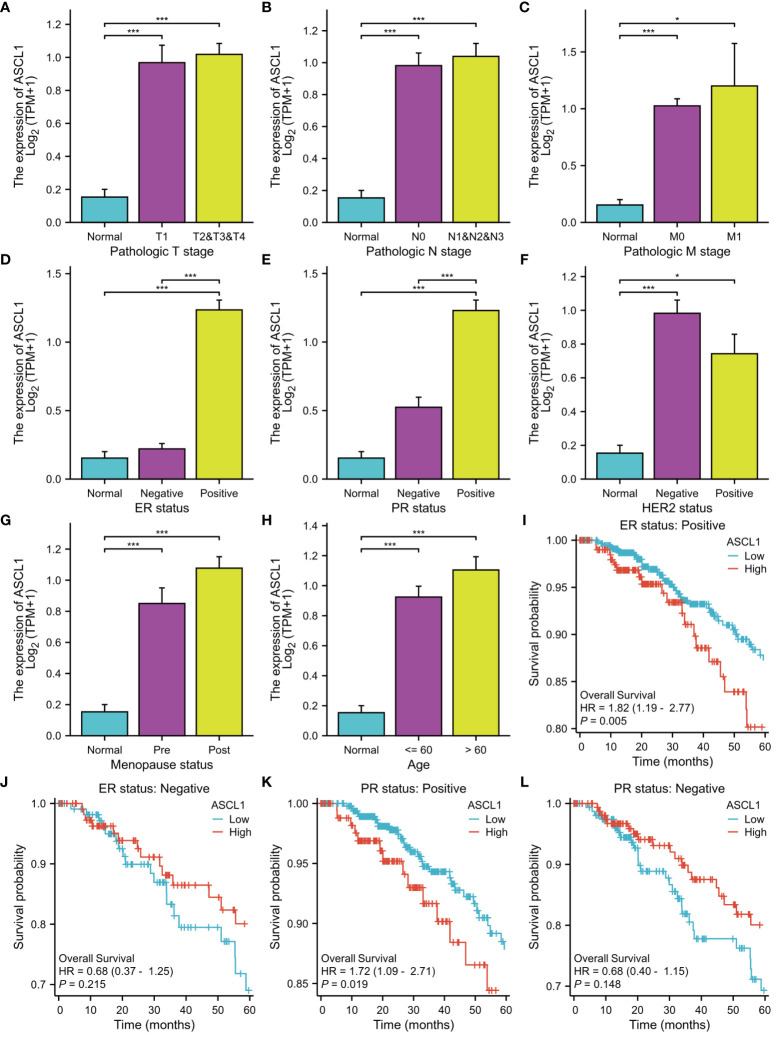
Clinical characteristics and prognosis are associated with *ASCL1* expression. The correlation between *ASCL1* expression and **(A)** pathologic T stage, **(B)** pathologic N stage, **(C)** pathologic M stage, **(D)** ER status, **(E)** PR status, **(F)** HER2 status, **(G)** menopause status, and **(H)** age. Overall survival was analyzed based on ER status [**(I)** ER-positive; **(J)** ER-negative] and PR status [**(K)** PR-positive; **(L)** PR-negative]. ^***^ *p* < 0.001; ^*^ *p* < 0.05. ER, estrogen receptor; PR, progesterone receptor; HER2, human epidermal growth factor receptor 2.

The expression of *ASCL1* was significantly correlated with clinical characteristics and immune cell infiltration. Analysis of the correlation between *ASCL1* expression and clinical characteristics revealed that factors, such as pathological T stage (**Figure 7A**), pathological N stage (**Figure 7B**), pathological M stage (**Figure 7C**), ER status (**Figure 7D**), progesterone receptor (PR) status (**Figure 7E**), HER2 status (**Figure 7F**), menopause status (**Figure 7G**), and age (**Figure 7H**), were associated with *ASCL1* expression. Compared with other clinical factors, *ASCL* expression was closely related to ER status and PR status. Therefore, ER status and PR status were used for stratified OS analysis. In ER-positive and PR-positive BC, *ASCL1* expression predicted poor prognosis (**Figures 7J, K**). However, *ASCL1* expression was not significantly correlated with the OS rate in ER-negative and PR-negative BC (**Figure 7I, L**).

Next, the correlation between the expression levels of genes and immune cell infiltration was examined. *ASCL1* expression was negatively correlated with the estimate (**Supplementary Figure S5A**), immune (**Supplementary Figure S5B**), and stromal scores (**Supplementary Figure S5C**). Analysis of the correlation between immune cell proportion and *ASCL1* expression revealed that ASCL1 expression was positively correlated with the proportion of eosinophils, Th2 cells, and NK CD56^bright^ cells (**Supplementary Figures S5D–F**) and negatively correlated with the proportion of neutrophils, macrophages, DCs, NK CD56^dim^ cells, B cells, and plasmacytoid DCs (pDCs) (**Supplementary Figures S5G–L**).

### ASCL1 contributes to chemotherapy resistance in BC

**Figure 8:**
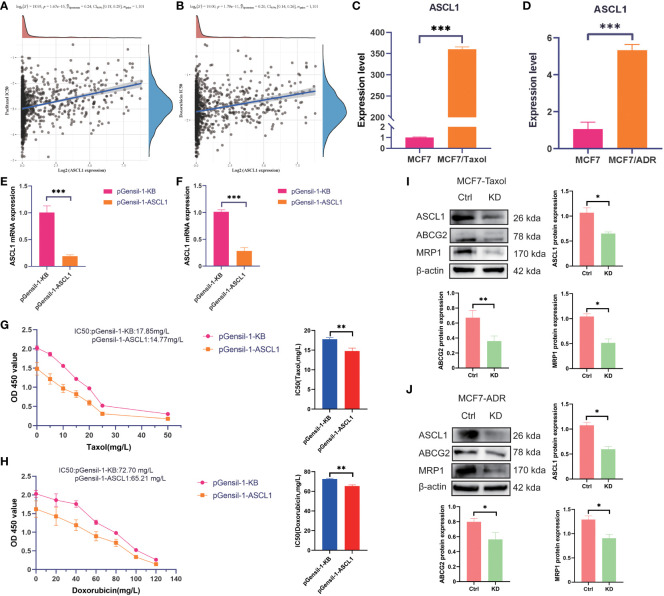
*ASCL1* confers chemotherapy resistance to breast cancer cells. Spearman’s analysis of the correlation between **(A)** *ASCL1* and paclitaxel, as well as between **(B)** ASCL1 and doxorubicin. qRT-PCR analysis of the expression of *ASCL1* between the following pairs: **(C)** MCF7 and MCF7-Taxol cells; **(D)** MCF7 and MCF7-ADR cells. The efficiency of *ASCL1* knockdown in **(E)** MCF7-Taxol and **(F)** MCF7-ADR cells. The correlation between *ASCL1* expression and taxol resistance in **(G)** MCF7-Taxol and **(H)** MCF7-ADR cells was examined using the CCK8 assay. Western blotting analysis of the correlation between ASCL1 and chemoresistance in **(I)** MCF7-Taxol and **(J)** MCF7-ADR cells. Ctrl, control group; KD, ASCL1 knockdown group; qRT-PCR, quantitative real-time polymerase chain reaction; CCK8, cell counting kit-8. MRP1: Another name for ABCC1. ***p < 0.001; **p < 0.01; *p < 0.05.

CRs are often closely related to drug sensitivity. *ASCL1* was the most DEG among the three clusters. To examine the correlation between *ASCL1* and chemotherapy resistance, the IC_50_ values of different drugs against different cell lines were obtained from the Genomics of Drug Sensitivity in Cancer database to perform correlation analysis. *ASCL1* expression was significantly correlated with resistance to paclitaxel and doxorubicin (**Figures 8A, B**). qRT-PCR analysis revealed that *ASCL1* expression in the MCF7-Taxol (**Figure 8C**) and MCF7-ADR (**Figure 8D**) cell lines was significantly higher than that in the MCF7 cell lines. Additionally, *ASCL1* was knocked down in MCF7-Taxol (**Figure 8E**) and MCF-ADR (**Figure 8F**) cell lines, which was confirmed using qRT-PCR analysis. The results of the CCK8 assay demonstrated that *ASCL1* knockdown significantly decreased the IC50 values of Taxol drugs against MCF7-Taxol (**Figure 8G**) and doxorubicin against MCF7-ADR (**Figure 8H**).

In this study, western blotting and qRT-PCR analyses were performed to examine the correlation between *ASCL1* and multiple drug resistance. Western blotting analysis demonstrated that ASCL1 knockdown downregulated the protein expression levels of ABCC1 and ABCG2 (**Figures 8I, J**). This indicates that *ASCL1* expression was significantly correlated with chemotherapy resistance in BC.

## Discussion

The identification of different tumor molecular subtypes is crucial for developing precision treatment and identifying novel therapeutic targets. Advances in molecular technology have revealed the role of CRs in tumor initiation and progression (22). However, limited studies have comprehensively analyzed the functional implications of CRs in BC.

This study identified different tumor molecular subtypes using differential analysis and Cox univariate analysis. Screening for prognostic-related CRs revealed the following 10 prognosis-related CRs: *ASCL1*, *BRCA2*, *CBX2*, *ERCC6L*, *PRDM12*, *PRDM16*, *PRMT8*, *RAD51*, *RAD54B*, and *UBE2T*. Furthermore, functional annotation using GO, KEGG, and PPI network analyses revealed that these genes are primarily involved in mitotic nuclear division processes and chromosome segregation and organization. To investigate the mechanisms contributing to heterogeneity in CR-related subtypes, different characteristics of the three clusters were analyzed. Previous studies have demonstrated that the tumor microenvironment (TME) encompasses stromal cells, immune cells coexisting with tumor cells and their secreted factors, vascular endothelial cells, and extracellular matrix (23). In the initial stage of tumor colonization or growth, the TME hinders the occurrence and development of tumors (24). However, the continuous stimulation of tumor antigens and immune activation responses results in the exhaustion or remodeling of the related effector cells in the TME, rendering them unable to perform physiological functions or promoting the malignant manifestations of tumors (25). The immune, stromal, and estimate scores were analyzed to evaluate the immune status of the three clusters. Cluster 3 exhibited the lowest immune, stromal, and estimate scores and decreased immune cell infiltration. These changes are associated with poor prognosis. Additionally, the differential expression of immune checkpoints and immunomodulatory factors between the three subtypes was analyzed. The expression levels of immune checkpoints and immunomodulatory factors varied between the three clusters, indicating the contribution of these factors to subtype heterogeneity.

Tumor heterogeneity, which is closely related to tumor progression and prognosis, was analyzed based on MATH, MSI, TMB, Neo, purity, and HRD scores (18). MATH effectively represented the deviation in minor allele frequency value distribution of specific mutation sites, demonstrating a positive correlation with tumor heterogeneity. Furthermore, TMB exhibits a strong correlation with the efficacy of PD-1/PD-L1 inhibitors. MSI, which results from impaired DNA mismatch repair in tumor tissues, serves as a crucial biomarker for identifying tumors with deficient DNA mismatch repair. Neoantigens, which arise from non-synonymous mutations and represent tumor-specific antigens, are potential immunotherapy targets owing to their abundant expression and enhanced immunogenicity in heterogeneous tumor cells. Moreover, tumor purity is significantly correlated with clinical characteristics, genomic expression, and biological characteristics of patients with tumors. HRD status, which serves as a crucial indicator for the treatment choice and prognosis of various tumors, is highly correlated with the sensitivity to platinum-based chemotherapy drugs and PARP inhibitors. In this study, MATH, TMB, purity, and HRD varied between the three subtypes. This indicated the presence of tumor heterogeneity, contributing to BC progression.

Advances in molecular and cancer biology have enabled the identification of various genomic, epigenomic, transcriptomic, and proteomic signatures that are correlated with cancer stemness. These molecular signatures were frequently linked to specific oncogenic signaling pathways that regulate transcriptional networks, which maintain cancer cell growth and proliferation (26). Transcriptional and epigenetic dysregulation of cancer cells promotes the acquisition of oncogenic dedifferentiation and stemness traits by altering signaling pathways involved in maintaining the physiological stem cell phenotype (27). Furthermore, the tumor stemness and mutation profiles of the three molecular subtypes were examined. The RNAss, EREG-EXPss, DNAss, and EREG-METHss varied between the three subtypes. Gene mutation in different clusters was analyzed. *TP53*, *PI3KCA*, *TTN*, *GATA3*, and *MUC16* were significantly mutated in the three subtypes. Mutations in tumor suppressor genes result in function loss and promote tumor progression.

The clinical characteristics are also related to the progression of cancer. Therefore, the clinical characteristics of the three clusters were examined. T stage was significantly correlated with the tumor molecular subtypes in the three subtypes. T1, stage 1, and luminal A subtypes accounted for a large proportion of cluster 1, which was correlated with a favorable prognosis. However, other clinical characteristics (N, M, and age) were not significantly correlated between the three clusters.

To further explore the differential gene expression patterns between groups and the related pathway changes, the three subtypes were subjected to pairwise differential analysis. *ASCL1* was identified as the most significantly upregulated gene and played a crucial role in regulating chromatin and epigenetic regulation. Additionally, ASCL1 is a key transcription factor (TF) in neuroendocrine tumors (28). ASCL1 is often enriched in transcriptionally active genomic regions of super-enhancers, which are characterized by a high density of TF binding sites and play a crucial role in modulating chromatin interactions with TFs during reprogramming (29). In this study, analysis of the correlation between CR and drug sensitivity demonstrated that chromatin status was correlated with anthracycline sensitivity in both ER-negative and ER-positive BC, as well as in both node-negative and node-positive BC (30).

Previous studies have demonstrated that ASCL1 reprograms prostate cancer by remodeling chromatin. Targeting ASCL1 can effectively change the phenotype of prostate cancer and mitigate drug resistance (31). ASCL1 confers osimertinib resistance to lung adenocarcinoma by initiating an EMT-related gene expression program in a permissible cellular environment (32). Additionally, ASCL1 plays a crucial role in neuroblastoma pathogenesis by promoting cell proliferation and differentiation (33). Mechanistic studies have revealed that ASCL1 can promote cAMP-response element binding protein (CREB) expression in prostate cancer. The phosphorylation of CREB promotes GPX4 transcription, inhibiting ferroptosis and conferring prostate cancer cells with resistance to androgen receptor antagonists (34). The CCK8 and qRT-PCR assay results demonstrated that *ASCL1* plays a major role in conferring chemotherapy resistance to BC cells.

ABCC1, a multidrug resistance protein, increases drug efflux by affecting the function of the ATP binding cassette (ABC) drug efflux pump, which is also named MRP1, contributing to multidrug resistance development in cancer cells (35). ABCG2, which is one of the members of ABC superfamily G, confers therapy resistance to cancer cells by promoting drug efflux (36). As ABCG2 is closely related to BC chemotherapy resistance, it is also called BC resistance protein. The correlation between ASCL1, ABCC1, and ABCG2 was examined using Western blotting. *ASCL1* knockdown downregulated the protein expression levels of ABCC1 and ABCG2 in MCF7-ADR and MCF7-Taxol cell lines. This indicated that ASCL1 is closely related to the chemosensitivity of BC.

This study identified the potential role of CRs in modulating the immune, mutation, and prognosis profiles of BC by dividing BC into different subtypes based on CRs. One study demonstrated that CRs regulate anthracycline sensitivity by modulating DNA accessibility in BC (30). Anthracycline and paclitaxel drugs are commonly used in BC chemotherapy and have important implications for BC treatment. Therefore, DEGs between different subtypes were analyzed to identify the core gene (*ASCL1*). Additionally, the effect of *ASCL1* on chemotherapy drug sensitivity was examined. The potential effects of CRs on the treatment of BC were clarified to provide a reference for identifying reliable targets and improving the clinical outcomes of patients with BC.

This study has some limitations that must be addressed in future studies. The construction and validation of the signature were dependent on the retrospective data from the TCGA database. Prospective studies using real-world data must be performed to assess the clinical feasibility of these molecular subtypes. Additionally, this study focused only on verifying the effect of ASCL1 on chemosensitivity. Further *in vitro* and *in vivo* experiments must be performed to elucidate the potential mechanism underlying the effect of ASCL1 on chemosensitivity.

## Conclusions

This study identified CR-associated subtypes of BC and comprehensively analyzed their clinical features, gene mutation status, immunophenotype, and drug sensitivity. These results provide a solid foundation for assessing clinical prognosis and developing personalized treatment strategies.

## Data availability statement

The original contributions presented in the study are included in the article/**Supplementary Material**. Further inquiries can be directed to the corresponding authors.

## Ethics statement

Ethical approval was not required for the studies on humans in accordance with the local legislation and institutional requirements because only commercially available established cell lines were used.

## Author contributions

YLL: Writing – original draft. XY: Writing – review & editing. CG: Writing – review & editing. YJL: Writing – review & editing. TT: Writing – review & editing. LZ: Writing – review & editing. FL: Writing – review & editing. MZ: Writing – review & editing. JH: Writing – review & editing. LM: Writing – review & editing.

## Supplementary material

Supplementary Figure S1 Heatmap of chromatin regulator-related DEGs between tumor and healthy tissues. DEGs, differentially expressed genes.

Supplementary Figure S2 Heatmap of chromatin regulator-related DEGs between different subtypes in the (A) TCGA-BRCA and (B) METABRIC cohort. DEGs, differentially expressed genes; TCGA-BRCA, The Cancer Genome Atlas–Breast Cancer; METABRIC, Molecular Taxonomy of Breast Cancer International Consortium.

Supplementary Figure S3 Expression of immune checkpoint-related and immunomodulator-related genes in different subtypes. **(A)** Summary of the expression of immune-checkpoint-related and immunomodulator-related genes in different subtypes. **(B)** Expression of immunomodulator inhibitor-related genes in the three subtypes. **(C)** Expression of MHC-related genes in the three subtypes. **(D)** Expression of receptor-related genes in the three subtypes. **(E)** Expression of immunomodulator stimulator-related genes in the three subtypes. **(F)** Expression of chemokine-related genes in the three subtypes. **(G)** Expression of immune checkpoint (inhibitor)-related genes in the three subtypes **(H)** Expression of immune checkpoint (stimulator)-related genes in the three subtypes. ^***^ *p* < 0.001; ^**^ *p* < 0.01; ^*^ *p* < 0.05. MHC, major histocompatibility complex.

Supplementary Figure S4 Clinical characteristics of different subtypes. **(A)** Total clinical characteristics of different subtypes. Chi-square test of the clinical characteristics of **(B)** age, **(C)** T stage, **(D)** N stage, **(E)** M stage, **(F)** tumor stage, and **(G)** tumor subtype in different subtypes. ^***^ *p* < 0.001; ^**^ *p* < 0.01; ^*^ *p* < 0.05.

Supplementary Figure S5
*ASCL1* expression is correlated with immune cell infiltration. The estimate method was used to evaluate the correlation of *ASCL1* expression with **(A)** estimate, **(B)** immune, and **(C)** stromal scores. Single-sample gene set enrichment analysis revealed that *ASCL1* expression was significantly correlated with the proportions of **(D)** eosinophils, **(E)** Th2 cells, **(F)** NK CD56^bright^ cells, **(G)** neutrophils, **(H)** macrophages, **(I)** DCs, **(J)** NK CD56^dim^ cells, **(K)** B cells, and **(L)** pDCs. Th2, T helper 2; NK, natural killer; DCs, dendritic cells; pDCs, plasmacytoid DCs.
