# Supplementary figures and images for: Identification of molecular subtypes based on chromatin regulator-related genes and experimental verification of the role of ASCL1 in conferring chemotherapy resistance to breast cancer
Source: Front Immunol. 2024 Apr 25;15:1390261. doi: 10.3389/fimmu.2024.1390261 (PMC11079216; doi:10.3389/fimmu.2024.1390261)

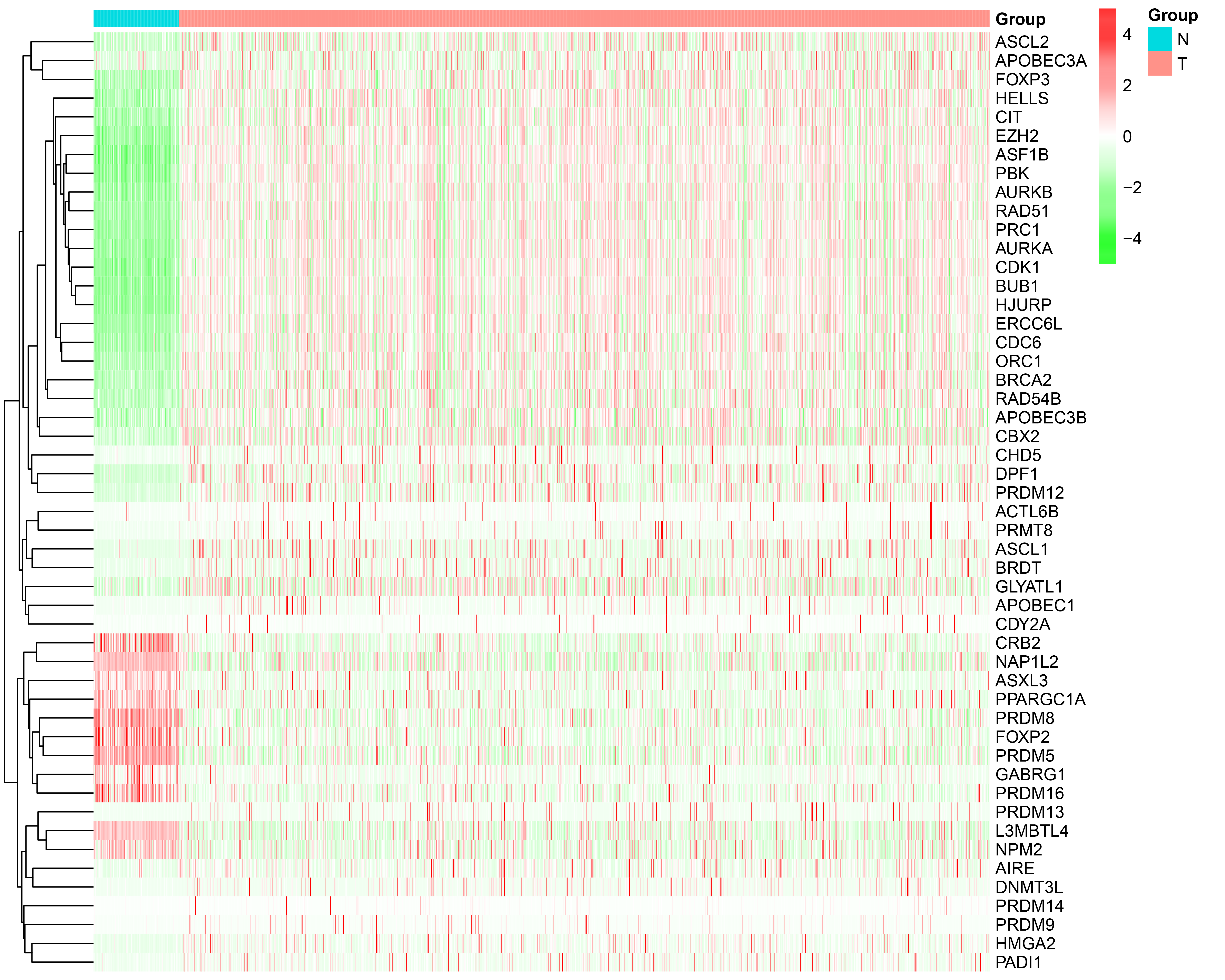

Supplement: Supplementary Figure S1 — Heatmap of chromatin regulator-related DEGs between tumor and healthy tissues. DEGs, differentially expressed genes. [file Image_1.tif]

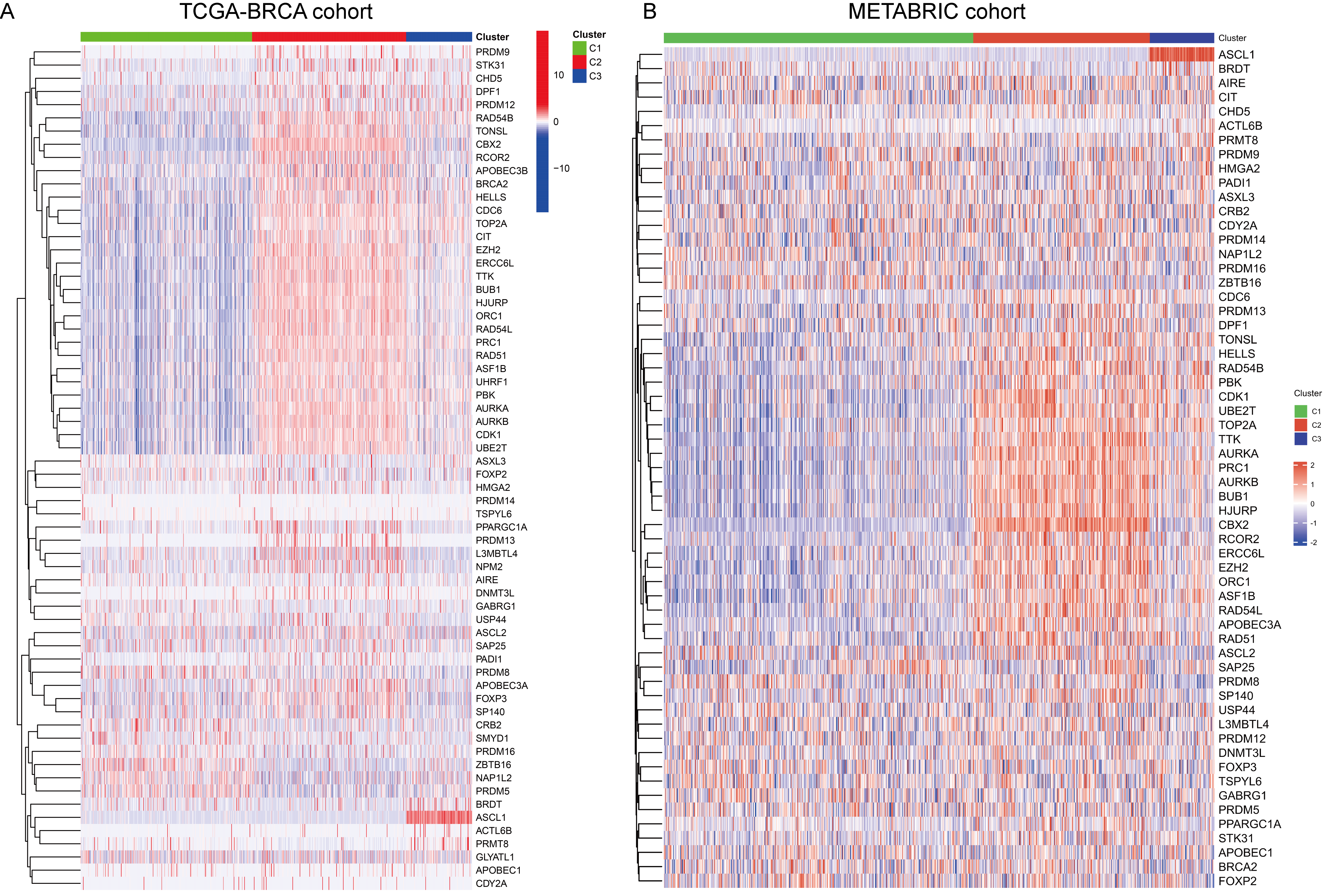

Supplement: Supplementary Figure S2 — Heatmap of chromatin regulator-related DEGs between different subtypes in the (A) TCGA-BRCA and (B) METABRIC cohort. DEGs, differentially expressed genes; TCGA-BRCA, The Cancer Genome Atlas–Breast Cancer; METABRIC, Molecular Taxonomy of Breast Cancer International Consortium. [file Image_2.tif]

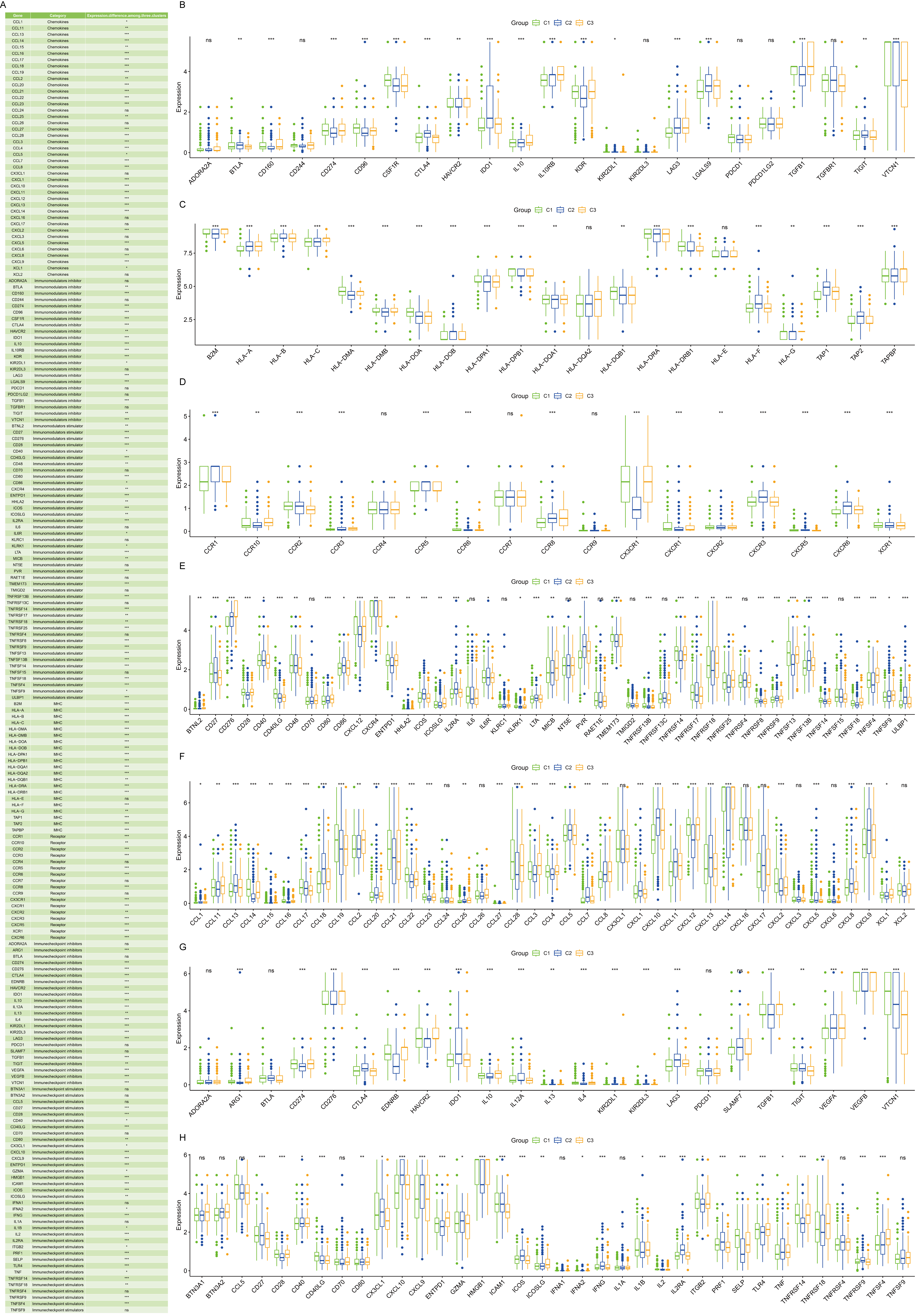

Supplement: Supplementary Figure S3 — Expression of immune checkpoint-related and immunomodulator-related genes in different subtypes. (A) Summary of the expression of immune-checkpoint-related and immunomodulator-related genes in different subtypes. (B) Expression of immunomodulator inhibitor-related genes in the three subtypes. (C) Expression of MHC-related genes in the three subtypes. (D) Expression of receptor-related genes in the three subtypes. (E) Expression of immunomodulator stimulator-related genes in the three subtypes. (F) Expression of chemokine-related genes in the three subtypes. (G) Expression of immune checkpoint (inhibitor)-related genes in the three subtypes (H) Expression of immune checkpoint (stimulator)-related genes in the three subtypes. *** p < 0.001; ** p < 0.01; * p < 0.05. MHC, major histocompatibility complex. [file Image_3.tif]

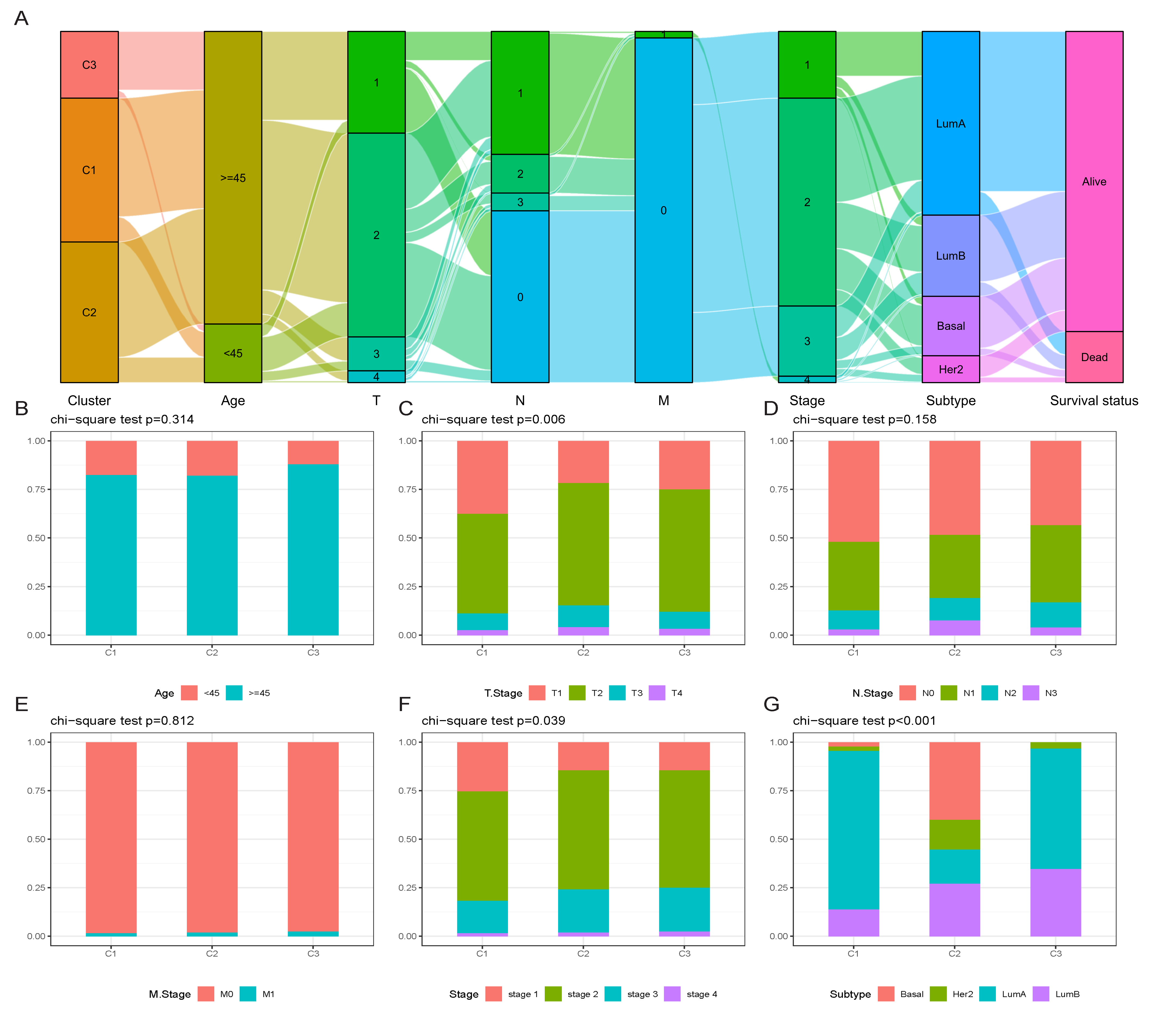

Supplement: Supplementary Figure S4 — Clinical characteristics of different subtypes. (A) Total clinical characteristics of different subtypes. Chi-square test of the clinical characteristics of (B) age, (C) T stage, (D) N stage, (E) M stage, (F) tumor stage, and (G) tumor subtype in different subtypes. *** p < 0.001; ** p < 0.01; * p < 0.05. [file Image_4.tif]

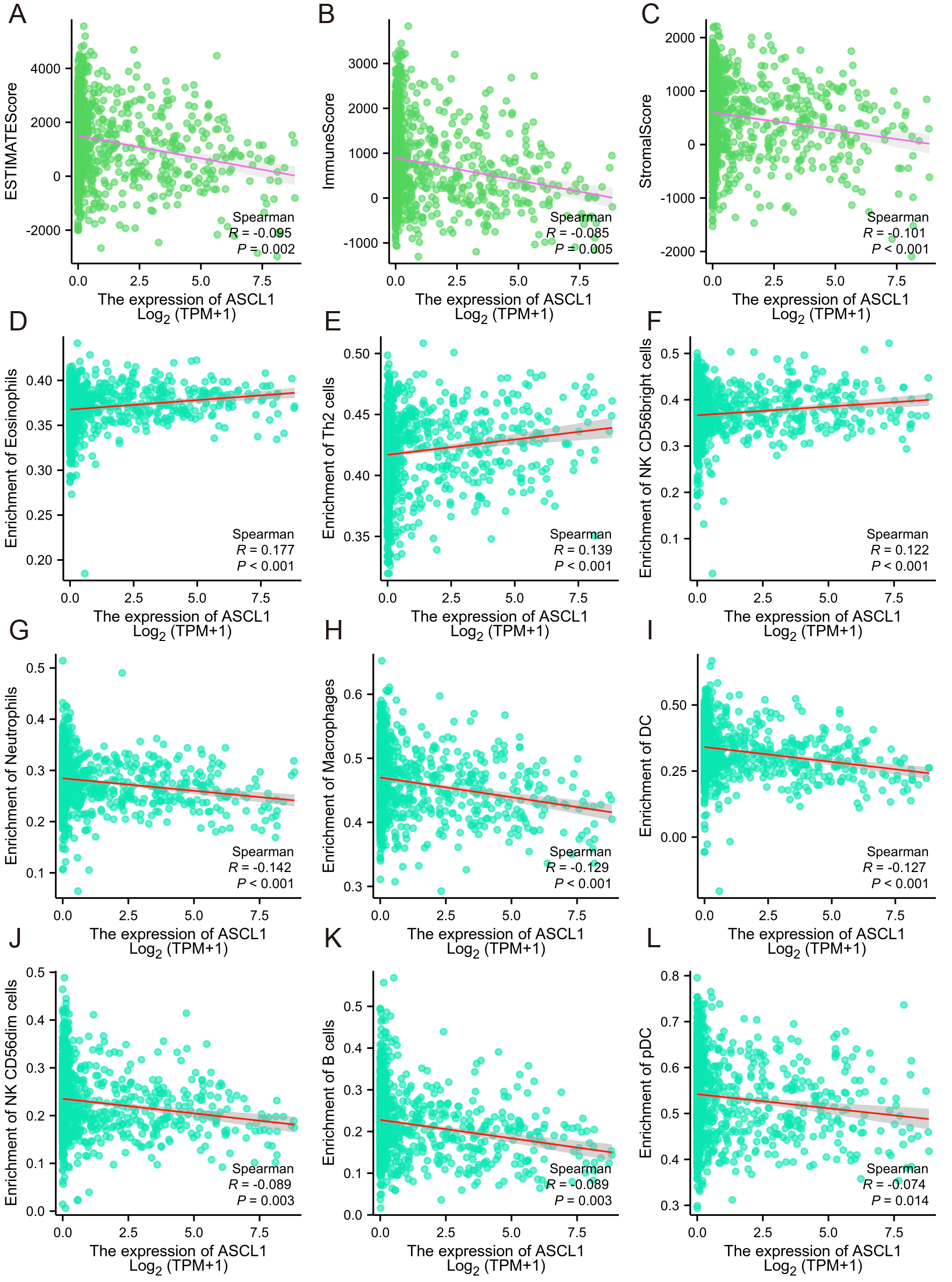

Supplement: Supplementary Figure S5 — ASCL1 expression is correlated with immune cell infiltration. The estimate method was used to evaluate the correlation of ASCL1 expression with (A) estimate, (B) immune, and (C) stromal scores. Single-sample gene set enrichment analysis revealed that ASCL1 expression was significantly correlated with the proportions of (D) eosinophils, (E) Th2 cells, (F) NK CD56bright cells, (G) neutrophils, (H) macrophages, (I) DCs, (J) NK CD56dim cells, (K) B cells, and (L) pDCs. Th2, T helper 2; NK, natural killer; DCs, dendritic cells; pDCs, plasmacytoid DCs. [file Image_5.tif]
